# The impact of shared decision-making on quality of life in systemic lupus erythematosus practice: findings from the TRUMP^2^-SLE prospective cohort study

**DOI:** 10.3389/fimmu.2026.1683992

**Published:** 2026-01-21

**Authors:** Chiharu Hidekawa, Nobuyuki Yajima, Ryusuke Yoshimi, Naoki Suzuki, Yuji Yoshioka, Natsuki Sakurai, Nao Oguro, Kenta Shidahara, Keigo Hayashi, Takanori Ichikawa, Dai Kishida, Yoshia Miyawaki, Ken-ei Sada, Yasuhiro Shimojima, Yuichi Ishikawa, Takaaki Komiya, Yosuke Kunishita, Daiga Kishimoto, Kaoru Takase-Minegishi, Yohei Kirino, Shigeru Ohno, Noriaki Kurita, Hideaki Nakajima

**Affiliations:** 1 Department of Stem Cell and Immune Regulation, Yokohama City University Graduate School of Medicine, Yokohama, Japan; 2 Division of Rheumatology, Department of Medicine, Showa Medical University School of Medicine, Tokyo, Japan; 3 Department of Healthcare Epidemiology, School of Public Health in the Graduate School of Medicine, Kyoto University, Kyoto, Japan; 4 Center for Innovative Research for Communities and Clinical Excellence, Fukushima Medical University, Fukushima, Japan; 5 Clinical Laboratory Department, Yokohama City University Hospital, Yokohama, Japan; 6 Department of Nephrology, Rheumatology, Endocrinology and Metabolism, Okayama University Graduate School of Medicine, Dentistry and Pharmaceutical Sciences, Okayama, Japan; 7 Department of Medicine (Neurology and Rheumatology), Shinshu University School of Medicine, Matsumoto, Japan; 8 Department of Clinical Epidemiology, Kochi Medical School, Kochi University, Nankoku, Japan; 9 The First Department of Internal Medicine, University of Occupational and Environmental Health, Kitakyushu, Japan; 10 Center for Rheumatic Disease, Yokohama City University Medical Center, Yokohama, Japan; 11 Department of Clinical Epidemiology, Graduate School of Medicine, Fukushima Medical University, Fukushima, Japan; 12 Department of Innovative Research and Education for Clinicians and Trainees, Fukushima Medical University Hospital, Fukushima, Japan

**Keywords:** lupus (SLE), non-health-related QoL, procreation, Quality of life (QoL), satisfaction with care, shared decision-making (SDM), systemic lupus erythematosus (SLE)

## Abstract

**Objectives:**

Shared decision-making (SDM) is increasingly emphasized in the treatment of systemic lupus erythematosus (SLE). Although SDM has been linked to quality of life (QoL) in various diseases, its direct and quantitative relationship with QoL in SLE remains unclear. This study aimed to investigate the longitudinal relationship between SDM and QoL in a multicenter cohort of patients with SLE.

**Methods:**

Patients aged 20 years or older diagnosed with SLE according to the 1997 revised American College of Rheumatology criteria were included. The association between baseline scores on the SDM-Q-9, an indicator of SDM, and one-year changes in scores on the LupusPRO, a QoL measure for patients with SLE, was examined using multiple regression analysis. Additionally, we evaluated the association between SDM changes and QoL, both overall and across the four groups, categorized by baseline SDM levels and SDM changes.

**Results:**

A total of 436 patients were included in this analysis. Higher baseline SDM-Q-9 scores were associated with a 0.16-point improvement (95% confidence interval [CI]: 0.071–0.24, *p* = 0.001) in non-health-related QoL, particularly in the satisfaction with care domain (0.36-point improvement, 95% CI: 0.14–0.58, *p* = 0.003). For health-related QoL, SDM-Q-9 scores improved the procreation domain by 0.13 points (95% CI: 0.033–0.22, *p* = 0.01*).* Longitudinal changes in SDM over one year did not substantially alter these associations.

**Conclusions:**

Higher SDM levels in patients with SLE may enhance their QoL. Sustained high SDM appears to be more influential than short-term improvements.

## Introduction

Shared decision-making (SDM) is a process in which healthcare providers and patients collaborate not only to comprehend diseases and therapeutic interventions but also to determine the optimal treatment approach based on patient values, physician experiences, and evidence (1). Although, the concept of SDM was proposed in 1972 (2), its adoption in clinical practice has only recently increased to improve medical care.

Systemic Lupus Erythematosus (SLE) is a chronic autoimmune disease characterized by the breakdown of immune tolerance and excessive production of autoantibodies. These autoantibodies form immune complexes that cause inflammation in various organs throughout the body. Recently, SDM has been recognized as an overarching principle in the context of SLE. Practicing treatment based on SDM between patients and healthcare providers is emphasized and explicitly stated in several recommendations (3–5). Moreover, patients with SLE have a reduced quality of life (QoL) compared with healthy individuals because of fatigue, pain, symptoms, and activity limitations (6–9). Importantly, previous studies have shown that patients consider QoL a key priority when making treatment decisions (10, 11). This finding suggests that insufficient attention to and incorporation of patient perspectives may lead to suboptimal treatment selection for QoL. Although the relationship between SDM and QoL has been explored in diseases such as bronchial asthma and malignant tumors (10, 12–15), the overall understanding of this relationship remains inconsistent (16, 17). Quantitative evidence directly linking SDM and QoL in patients with SLE remains limited.

In this study, we conducted a detailed analysis of the association between SDM and QoL in patients with SLE by utilizing data from the TRUMP^2^-SLE (Trust Measurement for Physicians and Patients with Systemic Lupus Erythematosus), a multicenter SLE cohort in Japan.

## Methods

### Study design and setting

This multicenter-cohort study was conducted using longitudinal data from the TRUMP^2^-SLE study. The TRUMP^2^-SLE study was conducted between June 2020 and August 2021 at five academic medical centers in Japan: Showa University Hospital, Okayama University Hospital, Shinshu University Hospital, Yokohama City University Hospital, and Yokohama City University Medical Center. Patients or the general public were not involved in the design or conduct of this study.

### Participants and data sources

Patients were included if they met the following criteria (1): aged 20 years or older, and (2) diagnosed according to the American College of Rheumatology (ACR) classification criteria revised in 1997 (18). Data were collected during routine outpatient follow-up visits, where patient background characteristics, disease activity, and patient-reported outcomes (SDM-Q-9 and LupusPRO) were obtained from annual questionnaires and medical records. Eligible patients attending the participating academic centers were consecutively approached by their attending rheumatologists during routine visits to minimize selection bias. Participation was voluntary, and written informed consent was obtained after the study procedures and purpose were explained. All participating rheumatologists in the study were Japanese, and physician data were collected using questionnaires.

### Exposure

Exposure was the SDM between the physician and patient, measured using the Japanese version of the SDM-Q-9 (19, 20). Patients completed the SDM-Q-9 during their annual outpatient follow-up visits. The SDM-Q-9 measures the degree of patient engagement in the decision-making process and has gained attention as a tool for measuring the dimensions of patient-centered care in rheumatology (21). The SDM-Q-9 comprises nine items covering agenda-setting, information sharing, deliberation, and decision-making, with input from both the patient and physician. Each item is rated on a six-point scale ranging from 0 (completely disagree) to 5 (completely agree). The total scores were derived by calculating the mean of each item and converting it to a 100-point scale, with higher values indicating better SDM. The Japanese version of the SDM-Q-9 has demonstrated high internal consistency, with a Cronbach’s alpha coefficient of 0.917, and its construct validity has also been established (20).

### Outcome measure

The outcome measure was QoL in patients with SLE, evaluated using the Japanese version of LupusPRO (22). The LupusPRO is a validated index consisting of 43 items broadly divided into two categories: health-related QoL (HRQoL) and non-health-related QoL (N-HRQoL). HRQoL was further divided into eight domains: SLE symptoms, cognitive function, medication concerns, pregnancy concerns, physical health, pain/vitality, psychological health, and body image. N-HRQoL was divided into four domains: future outlook, social support, stress coping, and medical satisfaction. The evaluation was conducted using a 5-point Likert scale ranging from 0 to 5: 0 = “none of the time,” 1 = “a little of the time,” 2 = “some of the time,” 3 = “most of the time,” 4 = “all of the time,” and 5 = “not applicable” (recoded as 0 for scoring). For items 1–34, “not applicable” was reverse-coded and treated as 4, following Elkaraly et al. The domain scores were calculated as the average of the points of each item, converted to a 100-point scale, with higher scores indicating better QoL (23). The category scores for both HRQoL and N-HRQoL were presented as the average of the domain scores included in each category. In the subsequent analyses, we defined the outcomes as one-year changes in LupusPRO scores, calculated as the differences between scores at one year and baseline for total and domain-specific scores.

### Covariate

Covariates were selected based on previous reports and the clinical perspectives of rheumatologists (7, 24–26). The variables included age, sex, duration of SLE, SELENA-Systemic Lupus Erythematosus Disease Activity Index (SELENA-SLEDAI), Systemic Lupus International Collaborating Clinics/American College of Rheumatology Damage Index (SDI), presence of therapeutic medications (glucocorticoids, immunosuppressants, and hydroxychloroquine [HCQ]), socioeconomic status (educational background, household income, and marital status), and baseline LupusPRO domain scores. To incorporate physician-related factors, the age and sex of the rheumatologists were included as covariates (26, 27). The SELENA-SLEDAI and SDI scores were assessed by the treating rheumatologist. Immunosuppressants were considered present if one or more of the following drugs were used: cyclophosphamide, mycophenolate mofetil, mizoribine, methotrexate, azathioprine, tacrolimus, cyclosporine, rituximab, or belimumab. Regarding marital status, having a spouse was defined regardless of cohabitation, whereas divorce, bereavement, and never married were considered as not having a spouse. Annual income was categorized as <2.5 million yen (approximately <23,000 US dollars at the exchange rate during the patient survey period), ≥2.5 and <5 million yen (approximately 23,000–45,000 US dollars), ≥5 and <10 million yen (approximately 45,000–91,000 US dollars), and ≥10 million yen (approximately ≥91,000 US dollars). Educational background was classified as up to elementary or middle school, up to high school or junior college, and up to university or graduate school.

### Statistical analysis

The characteristics of participating patients and physicians are presented as median values with interquartile range (IQR) for continuous variables, and as counts and percentages (%) for categorical variables. Continuous variables were compared using the Mann–Whitney *U* test, and categorical variables were compared using Fisher’s exact test. To examine the relationship between the SDM levels at enrollment and the change in QoL after one year, we analyzed the association between SDM-Q-9 scores and HRQoL, N-HRQoL, or the score of each of the 12 domains using general linear models. Next, we assessed the association between changes in the SDM-Q-9 and LupusPRO scores over one year. Finally, we categorized the patients into four groups based on baseline SDM-Q-9 scores (high or low) and the change in SDM-Q-9 scores over one year (increased or decreased). We then evaluated the association between changes in the SDM-Q-9 score of each group and changes in each domain of the LupusPRO over one year. All general linear models accounted for clustering by physicians and included the aforementioned covariates, along with the baseline values of the respective outcomes.

Missing data for exposure and covariates were imputed using multiple imputation methods, generating 100 imputed datasets. Statistical significance was set at *p* < 0.05. All statistical analyses were performed using Stata/SE (version 17.0; StataCorp, College Station, TX, USA) and SPSS V.28.0 (IBM, Armonk, NY, USA).

## Results

### Patient characteristics

Of the 524 patients enrolled in the TRUMP^2^-SLE cohort, 438 who completed the 1-year follow-up met the inclusion criteria. For each analysis, only patients whose attending physicians’ information was complete and had no missing values in the SDM-Q-9 and in the domain category of LupusPRO, which was the outcome of the analysis, were included, resulting in a total of 436 patients.

Characteristics of the patients who participated in this study are shown in the upper row of **Table 1**. The median age was 45.5 years (IQR 36.2–55.9), and 381 (87.4%) were female. The median SELENA-SLEDAI score, indicating disease activity in SLE, was 4 (IQR 1–6) and the median SDI, a measure of irreversible damage caused by SLE disease activity and its treatment, was 0 (IQR 0–1). Regarding treatment, approximately half of the patients were receiving glucocorticoids at doses less than 5 mg/day (prednisolone equivalent). Approximately 70% were taking some form of immunosuppressant, and approximately half were taking hydroxychloroquine. The median SDM-Q-9 score, indicating the extent of patient participation in SDM, was 77.8 (IQR 62.2–91.1). Patients’ HRQoL was 85.5 (IQR 72.4–92.9) and N-HRQoL was 43.5 (IQR 31.9–53.5) for the LupusPRO QoL measure.

**Table 1:** The characteristics of patients and rheumatologists at the baseline.

Variable	Value	Missing
Patient characteristics	(*n* = 436)	
Sex, female, n (%)	381 (87.4)	0
Age (years)^a^	45.5 (36.2-55.9)	0
SDM-Q-9 score^a^	77.8 (62.2-91.1)	19
HRQoL^b^	85.5 (72.4-92.9)	91
N-HRQoL^b^	43.5 (31.9-53.5)	74
BMI^a^	20.9 (19.3-23.5)	6
Disease duration (year)^a^	12.6 (6.5-20.0)	6
Clinical findings		
SELENA-SLEDAI score^a^	4 (1-6)	0
SDI score^a^	0 (0-1)	0
Drugs		
Glucocorticoid dose (mg/day)^a^	5 (2.5-7)	1
Immunosuppressants, n (%)^c^	302 (69.4)	1
Hydroxychloroquine, n (%)	216 (49.8)	2
Annual income		73
<2.5 million, n (%)	73 (20.1)	
≥2.5, <5 million yen, n (%)	112 (30.9)	
≥5, <10 million yen, n (%)	141 (38.8)	
≥10 million yen, n (%)	37 (10.2)	
Education		27
Up to elementary, middle school, n (%)	18 (4.4)	
Up to high school, junior college, n (%)	285 (69.7)	
Up to university, graduate school, n (%)	106 (25.9)	
Married state (single), n(%)	177 (42.7)	21
Sex match with one’s rheumatologist, n (%)	87 (20.0)	0
Rheumatologist characteristics	(*n* = 39)	
Age of rheumatologists(years)^a^	39 (34.5-43)	0
Sex of rheumatologists, female, n (%)	9 (23.1)	0

a Values are expressed as median (interquartile range).

b The domains of LupusPRO.

c At least one of the following: cyclophosphamide, mycophenolate mofetil, mizoribine, methotrexate, azathioprine, tacrolimus, cyclosporine, rituximab, or belimumab.

*HRQoL*, health-related QoL; *BMI*, Body Mass Index; *SELENA-SLEDAI*, SELENA-Systemic Lupus Erythematosus Disease Activity Index; *SDI*, Systemic Lupus International Collaborating Clinics/American College of Rheumatology Damage Index.

### Rheumatologist characteristics

The characteristics of the 39 rheumatologists responsible for treating the patients are shown in the lower rows of **Table 1**. The median age of the rheumatologists who participated in the study was 39 years, and 23.1% were female. Approximately 20% of the patients were of the same sex as the patient-rheumatologist combination.

### Association of baseline SDM-Q-9 scores changes in LupusPRO over one year

**Figure 1:**
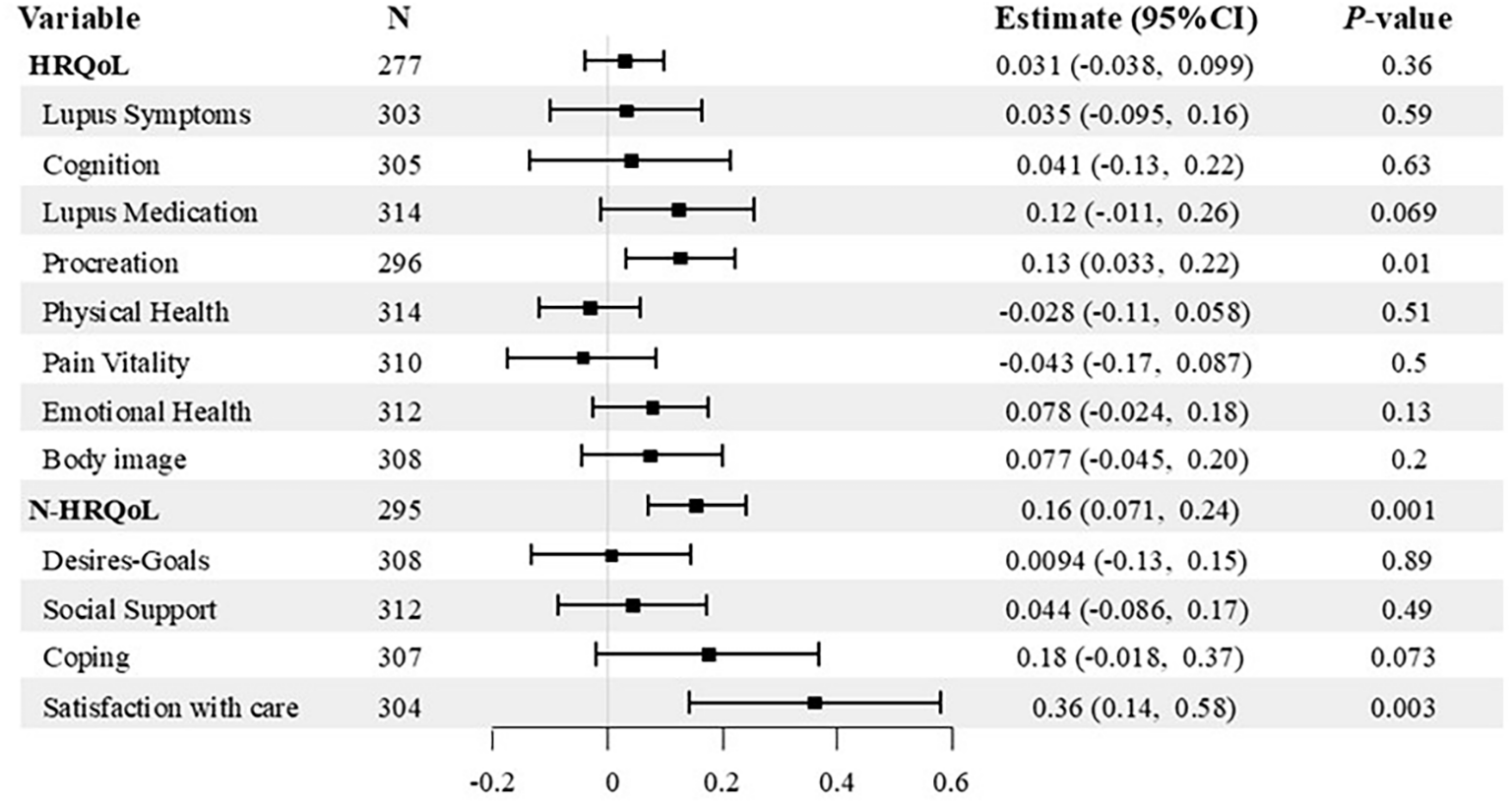
Forest plot showing associations between baseline SDM levels and 1-year changes in QOL. Associations between baseline SDM-Q-9 scores and 1-year changes in LupusPRO HRQoL, N-HRQoL, and each of the 12 domains were analyzed using general linear models. Black squares represent point estimates; error bars indicate 95% confidence intervals. Full results are available in **Supplementary Table 1**.

We conducted multiple regression analyses to investigate whether baseline SDM levels were associated with changes in QoL over the subsequent year. The results showed that a high SDM level contributed to an increase in N-HRQoL scores after one year (coefficient, 0.16 pts [95% CI 0.071 to 0.24], *p* = 0.001). Furthermore, analyzing each domain of N-HRQoL revealed that high SDM levels contributed to increases in “satisfaction with care” after one year (0.36 pts [95% CI 0.14 to 0.58], *p* = 0.003). Although no contribution of SDM level to the change in overall HRQoL score was found, a significant positive association was noted in the “procreation” domain (0.13 pts [95% CI 0.033 to 0.22], *p* = 0.01) (**Figure 1**; **Supplementary Table 1**).

### Association of change in SDM-Q-9 scores over one year with changes in LupusPRO

Although the SDM-Q-9 score did not significantly change over one year (0.0 [IQR -11.1–6.7], *n* = 401), some patients showed an increase in their scores while others showed a decrease. Therefore, we examined the association between changes in SDM-Q-9 scores over one year and changes in LupusPRO scores using multivariate analyses. The results showed that an increase in SDM-Q-9 scores contributed to positive changes in the domains of “body image” in HRQoL (0.14 pts [95% CI 0.025 to 0.26], *p* = 0.02) and “satisfaction with care” in N-HRQoL (0.20 pts [95% CI 0.063 to 0.33], *p* = 0.006). In contrast, an increase in SDM-Q-9 scores was not associated with improvement in the domain of “social support” in N-HRQoL (-0.10 pts [95% CI -0.20 to -0.0075], *p* = 0.036) (**Supplementary Figure 1**; **Supplementary Table 2**).

### Stratified sub-analysis by baseline score and one-year change in SDM-Q-9

Finally, we performed a stratified analysis based on baseline scores and changes in scores after one year for the SDM-Q-9. We used baseline SDM-Q-9 scores as a reference point to divide the participants into high- and low-scoring groups. Furthermore, within each group, the participants were divided into those whose SDM-Q-9 scores increased and those whose scores decreased after one year. This resulted in four groups: low baseline scores that decreased (LD group), low baseline scores that increased (LI group), high baseline scores that decreased (HD group), and high baseline scores that increased (HI group). The details of this classification are presented in **Supplementary Figure 2**. The LD group was used as the reference for comparison with the other three groups.

**Figure 2:**
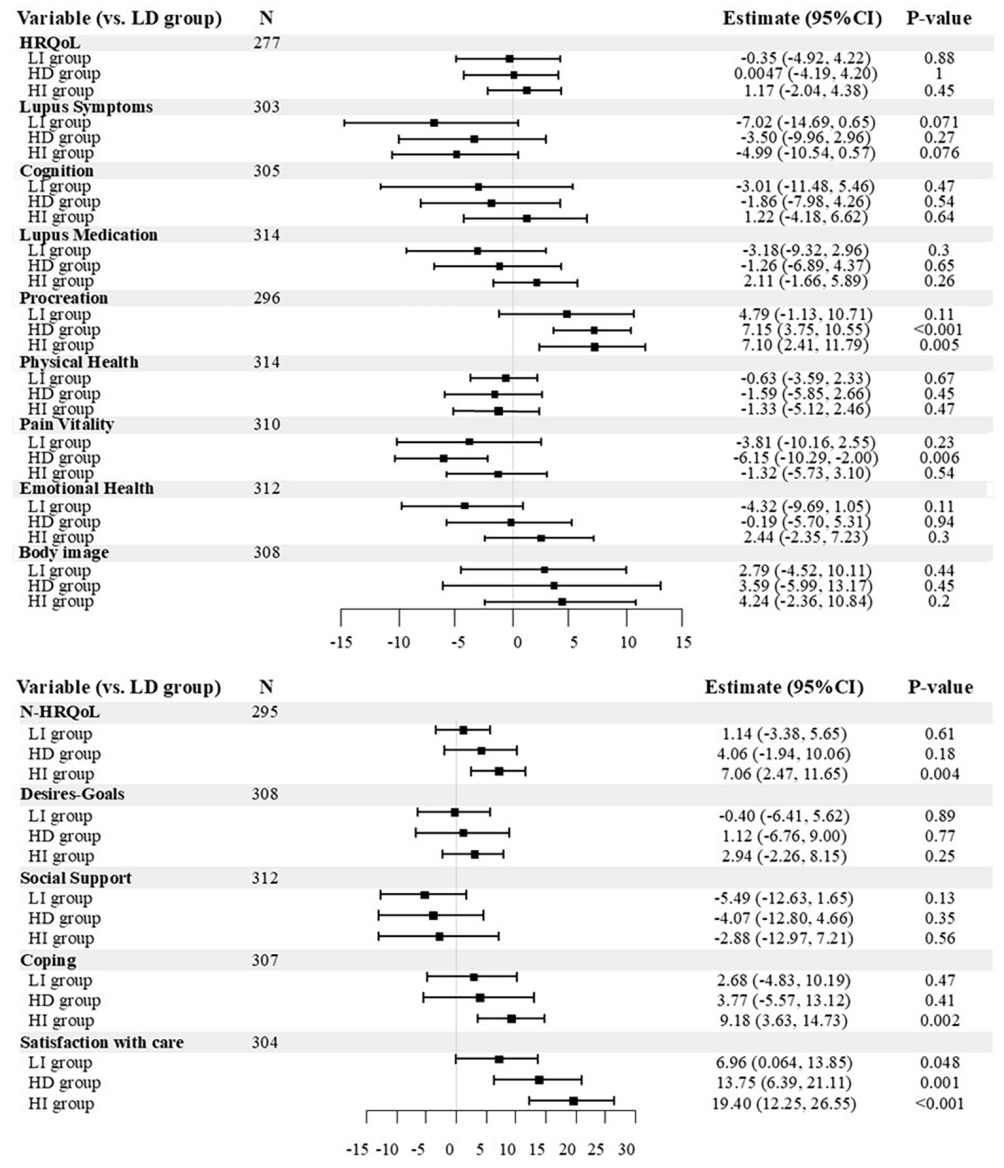
Forest plot of the impact of baseline levels and changes in SDM-Q-9 scores on LupusPRO. Patients were categorized into four groups based on baseline SDM-Q-9 scores (high or low) and changes in SDM-Q-9 scores over one year (increased or decreased). We analyzed the impact of these groups on LupusPRO using general linear models. This forest plot presents results for the four groups categorized by SDM-Q-9 scores. Full results are shown in **Supplementary Table 3**. LD, Decreasing SDM from Low Baseline; LI, Increasing SDM from Low Baseline; HD, Decreasing SDM from High Baseline; HI, Increasing SDM from High Baseline.

In the “satisfaction with care” domain, participants in the HD, HI, and LI groups showed a significantly greater positive impact compared to the LD group, regardless of baseline SDM-Q-9 levels or changes after one year (13.75 pts [95% CI 6.39 to 21.11], *p* = 0.001, 19.40 pts [95% CI 12.25 to 26.55], *p* < 0.001, and 6.96 pts [95% CI 0.064 to 13.85], *p* = 0.048, respectively). In the “procreation” domain, both the HD and HI groups exhibited a significantly greater positive impact, irrespective of SDM-Q-9 score changes (7.15 pts [95% CI 3.75 to 10.55], *p* < 0.001 and 7.10 pts [95% CI 2.41 to 11.79], *p* = 0.005, respectively). For overall N-HRQoL and the “coping” domain, a significant positive impact was observed in the HI group (7.06 pts [95% CI 2.47 to 11.65], *p* = 0.004 and 9.18 pts [95% CI 3.63 to 14.73], *p* = 0.002, respectively). Conversely, the contribution of SDM-Q-9 to the “pain vitality” domain was significantly lower in the HD group than that in the LD group (-6.15 pts [95% CI -10.29 to -2.00], *p* = 0.006). No significant effects similar to those observed in **Supplementary Figure 1** were found in the “body image” and “social support” domains in this analysis (**Figure 2**; **Supplementary Table 3**).

## Discussion

In this study, we examined the association of SDM with the QoL in patients with SLE using data from a multicenter longitudinal cohort study. Our findings indicate that favorable baseline SDM scores positively influenced QoL scores after one year. Additionally, improvements in SDM over one year contributed to positive longitudinal changes in certain aspects of QoL.

We divided the analysis into four groups based on baseline SDM levels and subsequent SDM changes because we considered that the impact of SDM changes on QoL might differ depending on baseline SDM levels. In fact, for example, in overall N-HRQoL and the coping domain, a significant positive effect of the SDM-Q-9 was observed in the HI group compared to the LD group, but not in the LI or HD groups. Therefore, it is suggested that the meaning of SDM changes may differ depending on the baseline SDM level. Patients with higher baseline SDM-Q-9 scores showed increased overall N-HRQoL scores, procreation domain scores, and satisfaction with care, regardless of changes in SDM-Q-9 scores over one year. Although quantitative reports examining factors related to the SDM-QoL association are limited, previous studies align with our findings in terms of satisfaction with care. Some previous studies have shown that SDM improves the trust relationship between SLE patients and their physicians (24, 28, 29). Indeed, we conducted a longitudinal analysis within this cohort, showing that higher baseline SDM-Q-9 scores in patients with SLE are associated with increased trust in their attending physicians and physicians in general at one-year (26). Moreover, the effects of SDM in patients with SLE include reducing decision-making conflict and improving patient knowledge (30–34). Furthermore, the QoL of patients with rheumatic diseases can be influenced by the nature of patient-physician interactions, with studies suggesting that the quality of communication with physicians is related to patient health outcomes (35). These findings suggest that SDM improves patient-healthcare provider communication, enhances understanding and agreement, respects patient autonomy, and facilitates discussions about treatment goals. As a result, treatments aligned with the patient’s values and life stages, thereby increasing their satisfaction with care. These improvements may explain the positive QoL outcomes observed in the present study.

Additionally, effective SDM may contribute to improvements in procreation. It remains unclear which patients are more likely to benefit from SDM in terms of enhancing their QoL, particularly regarding factors like sex, age, and disease activity. Our analyses revealed an association between SDM and procreation. The procreation domain of the LupusPRO reflects concerns regarding future pregnancies. While several studies have explored SDM in pregnant women, none have examined the association between SDM and future pregnancies. However, considering studies that suggest SDM reduces decision-making conflicts in pregnant women (36) and that a lack of SDM increases the risk of postpartum depression (37), SDM may have a positive effect on concerns regarding future pregnancy. Given that the population of this study was 87% female, and over half were of childbearing age, it is possible that sex and age influenced the impact on each QoL domain. Therefore, further investigation is needed to understand the effects of sex and age on SDM-QoL relationships.

However, improvements in SDM may not always lead to improvements in the QoL. In this study, the HD group showed a negative association between changes in SDM-Q-9 and pain vitality scores compared to the LD group. This finding may be due to decline in SDM reducing disease understanding, and decreasing awareness of SLE symptoms among groups with initially high SDM levels. Although previous studies have indicated that SDM improves patient knowledge, much of this knowledge focuses on treatment (30, 31, 33). Evidence on how SDM specifically influences the understanding of SLE symptoms and disease activity is lacking, and further investigation is required.

In this cohort, despite being treated at academic institutions, only about half of patients in this cohort were receiving HCQ. In Japan, historical safety concerns about chloroquine delayed HCQ approval for SLE compared with Europe and the United States, with approval granted only in 2015. Consequently, the introduction of HCQ among Japanese patients with SLE has lagged behind that in Western countries. As with the introduction of other medications, initiation of HCQ may be associated with both the exposure (SDM) and the outcome (QoL), potentially functioning as a confounder. Therefore, we incorporated HCQ use as a covariate into our analytical model to more accurately assess the association between SDM and QoL. Nonetheless, caution is warranted when generalizing our findings to countries with substantially different HCQ utilization patterns. Recent treatment guidelines from the Japan College of Rheumatology are largely aligned with international recommendations or guidelines, including those from EULAR and the ACR, and prescribing practices for HCQ in Japan have become more proactive in recent years. In our previous Japanese SLE cohort study, HCQ users were younger and had higher disease activity than non-users (38), likely reflecting a tendency to introduce HCQ earlier in newly diagnosed or relapsed patients. Therefore, Japanese society is not more restrictive than those of other countries in providing treatment that aligns with patients’ values and life stages. Given that younger patients and those of childbearing age tended to receive HCQ more frequently, the results for the procreation domain observed in this study may reasonably reflect trends applicable to patient populations in other countries.

Communication tools are useful for the effective and efficient implementation of SDM. Because SLE treatment is mainly conducted in outpatient settings, it is necessary to acquire and enhance deep SDM within a short period. In rheumatoid arthritis, several communication tools have been considered to contribute to the efficiency of SDM in clinical settings (30, 32, 39, 40). Although some reports have described communication tools for SDM in SLE (34, 41), no consensus has been reached, highlighting a challenge for future studies.

This study has two major strengths. First, it is the first study to report a direct quantitative relationship between SDM and QoL in patients with SLE. The practice of SDM in SLE is highlighted as one of the overarching principles in the recommendations of the European Alliance of Associations for Rheumatology (EULAR) (3–5), emphasizing the importance of making treatment decisions in collaboration with patients, not just healthcare providers. Unfortunately, research on SDM in patients with SLE is limited. Among the 61 papers published in the field of connective tissue diseases, only 11 (17%) focused on SLE (42). Furthermore, the association between SDM and QoL has not been thoroughly examined, indicating the need for further research. This longitudinal study shares the innovative characteristics of our project and provides new insights into the effects of SDM. Second, this is that no previous studies had incorporated rheumatologist-related factors in its investigation of the relationship between SDM and QoL. However, we previously reported, from the TRUMP^2^-SLE study, that difficulties in changing treatment goals by the attending rheumatologists are associated with lower Lupus low disease activity state (LLDAS) achievement in patients with SLE (43), highlighting the importance of considering physician factors concerning patient outcomes. By including rheumatologist-related factors in our analysis, we adjusted for their influence, providing a more comprehensive understanding of the SDM–QoL association.

The limitations of this study include the following. First, the patients included in our study were receiving outpatient treatment at large medical institutes, such as universities. Consequently, the cohort may have included patients with relatively high disease severity or complications who require care from multiple medical specialties other than rheumatology. Although even patients with stable SLE are often followed at large medical institutions in Japan, this cohort may still have differed from patients who undergo regular follow-ups at local clinics. However, considering the relatively low SLEDAI and SDI scores observed in this cohort, it is likely that many patients with stable disease were included in the analyzed population. Given this point, it remains unclear whether the results of this study can be generalized to patients attending medical institutions of all sizes, patients with any level of disease activity, or any situation involving SDM. Second, the study population consisted of Japanese residents, and the majority were Japanese. Therefore, many patients receive treatment according to the SLE treatment guidelines of the Japan College of Rheumatology (44). Consequently, the applicability of the findings from this study to regions outside Japan, with different cultures and healthcare systems, requires careful consideration. However, Japanese clinical guidelines have been developed in line with global recommendations and guidelines, such as ACR and EULAR, and the medical practices in Japan also extensively reference these international guidelines. Thus, apart from the cultural differences, the situation was similar. We plan to conduct international collaborative research to confirm these associations. Third, because many patients in this study had a long disease duration, those with higher SDM at baseline may have already achieved optimal QoL, reaching a ceiling effect. If this is the case, our study may have underestimated the impact of SDM on the QoL of patients with SLE. Fourth, although the construct validity of SDM-Q-9 has been demonstrated in previous studies involving patients with chronic diseases, it cannot be ruled out that the specific circumstances of SLE may have some impact on this validity. However, several SLE cohort studies have successfully applied the Japanese version of SDM-Q-9 and demonstrated theoretically consistent associations with physician factors, trust in physicians, and online information-seeking behavior (26, 45, 46). These findings provide indirect support for its construct validity in SLE populations. Furthermore, since the SDM concept transcends individual disease categories and broadly reflects the decision-making process between patients and physicians, we consider the practical limitations of applying this measure to be minimal. Finally, neither a validated minimal clinically important difference (MCID) nor cross-sectional thresholds have been established for the SDM-Q-9 scale at present, making it difficult to determine what constitutes a clinically meaningful change. Similarly, although LupusPRO is widely used in SLE research, no validated MCIDs or cross-sectional thresholds have been established for its individual domains, which limits the interpretability of the magnitude of change observed relative to SDM scores. Under these circumstances, we classified patients simply according to whether their SDM-Q-9 score increased or decreased in the stratified analysis. However, this approach may have led to patients with clinically insignificant changes being assigned to each group, potentially obscuring the relationship between the SDM-Q-9 and LupusPRO domains. Despite this concern, it is important to note that the baseline QoL scores of the participants showed variability, and significant improvements were observed in certain domains, even with a long disease duration. This suggests that there was still room for improvement in the QoL of many patients. Future research is expected to establish either an MCID or a clinically interpretable threshold for both the SDM-Q-9 and LupusPRO.

In conclusion, high-quality SDM in patients with SLE may contribute to improved QoL. SDM can alleviate pregnancy concerns and enhance satisfaction with healthcare. Notably, maintaining good SDM over time may have a more substantial impact on QoL than short-term SDM improvements. Therefore, effective communication tools for improving SDM in patients with SLE should be developed and utilized in clinical practice in the near future.

## Data Availability

The raw data supporting the conclusions of this article will be made available by the authors, without undue reservation.
