# Supplementary material for: The impact of shared decision-making on quality of life in systemic lupus erythematosus practice: findings from the TRUMP2-SLE prospective cohort study
Source: Front Immunol. 2026 Jan 21;17:1683992. doi: 10.3389/fimmu.2026.1683992 (PMC12868130; doi:10.3389/fimmu.2026.1683992)
Supplement: Supplementary file 1 [file DataSheet1.docx]

**Supplemental Table1** **Full Data on Comparison of changes in LupusPRO domain after 1 year with baseline SDM-Q-9 score**

| **Variable** | **HRQoL**  **(n=277)** | | | **Lupus Symptoms**  **(n=303)** | | | | **Cognition**  **(n=305)** | | | | **Lupus Medication**  **(n=314)** | | **Procreation**  **(n=296)** | | **Physical Health**  **(n=314)** | | **Pain Vitality**  **(n=310)** | | **Emotional Health**  **(n=312)** | | **Body image**  **(n=308)** | |  |
| --- | --- | --- | --- | --- | --- | --- | --- | --- | --- | --- | --- | --- | --- | --- | --- | --- | --- | --- | --- | --- | --- | --- | --- | --- |
|  | **β[95%CI]** | **P-value** | | **β[95%CI]** | | **P-value** | | **β[95%CI]** | | **P-value** | | **β[95%CI]** | **P-value** | **β[95%CI]** | **P-value** | **β[95%CI]** | **P-value** | **β[95%CI]** | **P-value** | **β[95%CI]** | **P-value** | **β[95%CI]** | **P-value** | |
| Baseline SDM-Q-9 score | 0.031 [-0.038 to 0.099] | 0.36 | | 0.035 [-0.095 to 0.16] | | 0.59 | | 0.041 [-0.13 to 0.22] | | 0.63 | | 0.12 [ -0.011 to 0.26] | 0.069 | 0.13 [0.033 to 0.22] | 0.01 | -0.028 [-0.11 to 0.058] | 0.51 | -0.043 [-0.17 to 0.087] | 0.5 | 0.078 [-0.024 to 0.18] | 0.13 | 0.077 [-0.045 to 0.20] | 0.2 | |
| Age at baseline (years) | 0.026 [-0.12 to 0.17] | 0.71 | | -0.013 [-0.16 to 0.14] | | 0.86 | | 0.068 [-0.19 to 0.32] | | 0.59 | | -0.0093 [-0.20 to 0.18] | 0.92 | 0.30 [0.095 to 0.50] | 0.006 | 0.042 [-0.044 to 0.13] | 0.32 | -0.11 [-0.27 to 0.053] | 0.18 | -0.073 [-0.25 to 0.10] | 0.4 | -0.14 [-0.43 to 0.14] | 0.31 | |
| Sex (female) | -0.052 [-3.67 to 3.57] | 0.98 | | -1.93 [-7.54 to 3.68] | | 0.48 | | -1.65 [-8.99 to 5.69] | | 0.65 | | 3.10 [-4.35 to 10.55] | 0.4 | -0.0047 [-5.97 to 5.96] | 1 | -3.64 [-9.09 to 1.81] | 0.18 | 1.57 [-4.24 to 7.38] | 0.58 | -0.46 [-5.95 to 5.03] | 0.86 | -3.66 [-9.72 to 2.41] | 0.22 | |
| Disease duration (year) | 0.070 [-0.072 to 0.21] | 0.31 | | -0.018 [-0.24 to 0.20] | | 0.87 | | -0.0090 [-0.29 to 0.27] | | 0.95 | | 0.22 [-0.12 to 0.57] | 0.19 | -0.0038 [-0.17 to 0.16] | 0.96 | 0.033 [-0.19 to 0.26] | 0.76 | 0.11 [-0.18 to 0.40] | 0.44 | 0.081 [-0.17 to 0.33] | 0.51 | 0.16 [-0.096 to 0.40] | 0.21 | |
| SLEDAI | -0.16 [ -0.63 to 0.31] | 0.48 | | -0.11 [-0.80 to 0.59] | | 0.75 | | -0.27 [-1.14 to 0.61] | | 0.53 | | -0.40 [-1.23 to 0.43] | 0.33 | 0.074 [-0.31 to 0.46] | 0.7 | -0.11 [-0.45 to 0.23] | 0.52 | 0.024 [-0.48 to 0.53] | 0.92 | -0.52 [-1.19 to 0.15] | 0.12 | 0.13 [-0.38 to 0.64] | 0.61 | |
| SDI | -0.45 [-1.52 to 0.62] | 0.39 | | -1.03 [-3.02 to 0.95] | | 0.29 | | 0.81 [-2.00 to 3.63] | | 0.56 | | -2.98 [-4.81 to -1.15] | 0.003 | 0.17 [-1.01 to 1.34] | 0.77 | -1.42 [-2.32 to -0.52] | 0.003 | -0.23 [-1.46 to 1.00] | 0.7 | -0.50 [-2.18 to 1.19] | 0.55 | -0.76 [-2.39 to 0.86] | 0.34 | |
| Glucocorticoid dose (mg/day) | 0.095 [-0.37 to 0.56] | 0.67 | | 0.30 [-0.30 to 0.91] | | 0.3 | | -0.27 [-0.73 to 0.20] | | 0.25 | | -0.092 [-0.66 to 0.47] | 0.74 | 0.089 [-0.37 to 0.55] | 0.69 | 0.17 [-0.13 to 0.46] | 0.26 | -0.045 [-0.55 to 0.46] | 0.85 | 0.17 [-0.46 to 0.81] | 0.58 | -0.15 [-0.70 to 0.39] | 0.57 | |
| Immunosuppressants^a^ | -1.49 [-3.76 to 0.77] | 0.18 | | -0.22 [-6.02 to 5.58] | | 0.94 | | -1.78 [-6.60 to 3.04] | | 0.45 | | -1.69 [-8.81 to 5.43] | 0.63 | -1.77 [-5.70 to 2.16] | 0.36 | -1.56 [-5.86 to 2.74] | 0.46 | -0.25 [-5.13 to 4.64] | 0.92 | -2.69 [-6.98 to 1.59] | 0.21 | -5.87 [-10.46 to -1.27] | 0.015 | |
| Hydroxychloroquine | 1.25 [-0.18 to 2.67] | 0.084 | | 2.37 [-2.56 to 7.31] | | 0.33 | | 1.27 [-2.55 to 5.09] | | 0.5 | | 1.98 [-3.07 to 7.03] | 0.42 | -1.25 [-4.90 to 2.40] | 0.49 | 4.64 [2.22 to 7.06] | 0.001 | 0.59 [-2.93 to 4.12] | 0.73 | -2.35 [-5.96 to 1.26] | 0.19 | -3.49 [-8.60 to 1.62] | 0.17 | |
| Married state | 0.89 [-3.48 to 5.26] | 0.67 | | 1.73 [-5.09 to 8.54] | | 0.6 | | 1.07 [-6.01 to 8.16] | | 0.76 | | 2.46 [-5.93 to 10.85] | 0.55 | 0.17 [-4.98 to 5.32] | 0.95 | 3.49 [-0.50 to 7.29] | 0.084 | 1.93 [-2.83 to 6.70] | 0.41 | -1.97 [-6.09 to 2.15] | 0.33 | -2.40 [-7.71 to 2.92] | 0.36 | |
| Annual income  (vs. <2.5 million yen) | Ref. |  | | Ref. | |  | | Ref. | |  | | Ref. |  | Ref. |  | Ref. |  | Ref. |  | Ref. |  | Ref. |  | |
| ≥2.5, <5 million yen | 4.32 [0.22 to 8.43] | 0.04 | | 4.57 [-2.80 to 11.93] | | 0.21 | | 11.28 [4.19 to 18.36] | | 0.004 | | 3.08 [-4.30 to 10.45] | 0.39 | 5.82 [-1.83 to 13.47] | 0.13 | -0.74 [-6.04 to 4.55] | 0.77 | 2.56 [-5.70 to 10.82] | 0.52 | 7.51 [1.36 to 13.66] | 0.019 | 5.25 [-2.13 to 12.63] | 0.15 | |
| ≥5, <10 million yen | 5.20 [1.55 to 8.85] | 0.008 | | 4.25 [-3.17 to 11.67] | | 0.24 | | 10.99 [3.99 to 17.99] | | 0.004 | | 7.65 [-0.33 to 15.62] | 0.059 | 6.30 [-1.19 to 13.79] | 0.095 | 3.25 [-1.58 to 8.08] | 0.17 | 3.14 [-3.79 to 10.07] | 0.35 | 9.17 [3.26 to 15.09] | 0.004 | 3.37 [-5.33 to 12.07] | 0.43 | |
| ≥10 million yen | 6.64 [2.33 to 10.96] | 0.005 | | 5.07 [-3.42 to 13.56] | | 0.23 | | 13.79 [5.87 to 21.71] | | 0.002 | | 9.38 [-1.82 to 20.58] | 0.096 | 3.79 [-4.77 to 12.34] | 0.37 | 2.52 [-1.92 to 6.96] | 0.25 | 4.03 [-5.23 to 13.28] | 0.37 | 15.57 [6.33 to 24.81] | 0.002 | 2.71 [-6.07 to 11.49] | 0.53 | |
| Education  (vs. up to elementary, middle school) | Ref. |  | | Ref. | |  | | Ref. | |  | | Ref. |  | Ref. |  | Ref. |  | Ref. |  | Ref. |  | Ref. |  | |
| Up to high school, junior college | -0.77 [-9.43 to 7.89] | 0.86 | | -3.25 [-17.07 to 10.57] | | 0.63 | | 5.53 [-11.60 to 22.65] | | 0.51 | | -3.68 [-20.28 to 12.93] | 0.65 | -3.92 [-22.08 to 14.25] | 0.66 | -1.56 [-11.17 to 8.05] | 0.74 | -2.83 [-9.66 to 4.00] | 0.4 | -2.22 [-12.71 to 8.28] | 0.67 | 9.95 [-5.88 to 25.77] | 0.21 | |
| Up to university, graduate school | -2.27 [-11.32 to 6.79] | 0.61 | | -3.74 [-18.59 to 11.10] | | 0.6 | | 2.40 [-15.66 to 20.45] | | 0.79 | | -4.25 [-22.15 to 13.65] | 0.63 | -2.16 [-20.17 to 15.84] | 0.81 | -4.74 [-13.86 to 4.37] | 0.29 | -7.20 [-16.05 to 1.64] | 0.11 | -6.78 [-16.63 to 3.08] | 0.17 | 5.07 [-10.04 to 20.18] | 0.49 | |
| Factors of doctor |  |  | |  | |  | |  | |  | |  |  |  |  |  |  |  |  |  |  |  |  | |
| Dr. age | -0.22 [-0.44 to -0.0063] | 0.044 | | -0.25 [-0.69 to 0.19] | | 0.25 | | -0.47 [-0.97 to 0.034] | | 0.066 | | -0.41 [-1.04 to 0.22] | 0.19 | 0.022 [-0.41 to 0.46] | 0.92 | 0.0011 [-0.20 to 0.20] | 0.99 | -0.40 [-0.77 to -0.029] | 0.036 | -0.20 [-0.53 to 0.12] | 0.2 | -0.044 [-0.67 to 0.58] | 0.88 | |
| Dr. sex (female) | 5.67 [2.10 to 9.24] | 0.004 | | 6.50 [-0.47 to 13.48] | | 0.066 | | 10.12 [3.16 to 17.08] | | 0.006 | | 7.45 [-2.97 to 17.88] | 0.15 | -0.68 [-4.57 to 3.22] | 0.72 | 6.08 [3.46 to 8.70] | <0.001 | 6.54 [1.52 to 11.56] | 0.013 | 3.90 [-1.49 to 9.30] | 0.15 | 4.44 [-6.17 to 15.06] | 0.4 | |
| Baseline subdomain | -0.29 [-0.37 to -0.21] | <0.001 | | -0.42 [-0.60 to -0.25] | | <0.001 | | -0.47 [-0.58 to -0.36] | | <0.001 | | -0.52 [-0.64 to -0.40] | <0.001 | -0.46 [-0.63 to -0.28] | <0.001 | -0.37 [-0.51 to -0.23] | <0.001 | -0.39 [-0.47 to -0.32] | <0.001 | -0.28 [-0.39 to -0.17] | <0.001 | -0.35 [-0.43 to -0.28] | <0.001 | |
|  |  |  | |  | |  | |  | |  | |  |  |  |  |  |  |  |  |  |  |  |  | |
| **Variable** | **N-HRQoL**  **(n=295)** | | | | **Desires-Goals**  **(n=308)** | | | | **Social Support**  **(n=312)** | | | **Coping**  **(n=307)** | | **Satisfaction with care**  **(n=304)** | |  |  |  |  |  |  |  |  |  |
|  | **β[95%CI]** | | **P-value** | | **β[95%CI]** | | **P-value** | | **β[95%CI]** | | **P-value** | **β[95%CI]** | **P-value** | **β[95%CI]** | **P-value** |  |  |  |  |  |  |  |  |  |
| Baseline SDM-Q-9 score | 0.16 [0.071 to 0.24] | | 0.001 | | 0.0094 [-0.13 to 0.15] | | 0.89 | | 0.044 [-0.086 to 0.17] | | 0.49 | 0.18 [-0.018 to 0.37] | 0.073 | 0.36 [0.14 to 0.58] | 0.003 |  |  |  |  |  |  |  |  |  |
| Age at baseline (years) | 0.022 [-0.099 to 0.14] | | 0.71 | | -0.020 [-0.20 to 0.16] | | 0.81 | | -0.0086 [-0.21 to 0.19] | | 0.93 | 0.23 [0.033 to 0.43] | 0.025 | -0.16 [-0.68 to 0.37] | 0.54 |  |  |  |  |  |  |  |  |  |
| Sex (female) | -0.69 [-4.68 to 3.31] | | 0.73 | | 0.87 [-5.71 to 7.45] | | 0.79 | | 5.37 [-0.61 to 11.36] | | 0.076 | -2.12 [-9.06 to 4.82] | 0.53 | -7.62 [-20.37 to 5.12] | 0.23 |  |  |  |  |  |  |  |  |  |
| Disease duration (year) | 0.065 [-0.064 to 0.19] | | 0.31 | | 0.12 [-0.15 to 0.39] | | 0.37 | | -0.19 [-0.43 to 0.056] | | 0.13 | -0.11 [-0.33 to 0.10] | 0.3 | 0.30 [0.023 to 0.57] | 0.035 |  |  |  |  |  |  |  |  |  |
| SLEDAI | 0.080 [-0.31 to 0.47] | | 0.68 | | -0.24 [-0.66 to 0.18] | | 0.24 | | -0.22 [-0.93 to 0.49] | | 0.53 | 0.40 [-0.54 to 1.34] | 0.39 | 0.44 [-0.19 to 1.08] | 0.16 |  |  |  |  |  |  |  |  |  |
| SDI | -0.35 [-1.37 to 0.67] | | 0.49 | | 0.12 [-1.62 to 1.86] | | 0.89 | | 1.26 [-1.20 to 3.73] | | 0.3 | -0.56 [-2.04 to 0.91] | 0.44 | -1.60 [-3.75 to 0.56] | 0.14 |  |  |  |  |  |  |  |  |  |
| Glucocorticoid dose (mg/day) | -0.044 [-0.22 to 0.133] | | 0.61 | | 0.30 [-0.34 to 0.93] | | 0.35 | | -0.44 [-0.83 to -0.055] | | 0.027 | 0.024 [-0.45 to 0.50] | 0.92 | 0.11 [-0.17 to 0.38] | 0.43 |  |  |  |  |  |  |  |  |  |
| Immunosuppressants^a^ | 2.35 [0.35 to 4.35] | | 0.024 | | -1.05 [-5.88 to 3.78] | | 0.66 | | 2.63 [-1.86 to 7.11] | | 0.24 | 0.91 [-2.37 to 4.19] | 0.57 | 5.56 [0.29 to 10.84] | 0.04 |  |  |  |  |  |  |  |  |  |
| Hydroxychloroquine | -0.36 [-3.08 to 2.35] | | 0.79 | | 0.25 [-2.95 to 3.45] | | 0.87 | | 0.39 [-4.55 to 5.33] | | 0.87 | 1.69 [-6.97 to 10.35] | 0.69 | -4.24 [-9.04 to 0.56] | 0.08 |  |  |  |  |  |  |  |  |  |
| Married state | -0.99 [-4.18 to 2.21] | | 0.53 | | 3.58 [-2.73 to 9.88] | | 0.25 | | -1.74 [-7.04 to 3.55] | | 0.5 | 0.70 [-4.53 to 5.93] | 0.78 | -5.16 [-12.92 to 2.61] | 0.18 |  |  |  |  |  |  |  |  |  |
| Annual income  (vs. <2.5 million yen) | Ref. | |  | | Ref. | |  | | Ref. | |  | Ref. |  | Ref. |  |  |  |  |  |  |  |  |  |  |
| ≥2.5, <5 million yen | -1.12 [-5.12 to 2.88] | | 0.56 | | 1.06 [-7.23 to 9.34] | | 0.79 | | -3.78 [-12.00 to 4.43] | | 0.35 | -2.45 [-9.09 to 4.20] | 0.45 | 2.09 [-5.57 to 9.74] | 0.57 |  |  |  |  |  |  |  |  |  |
| ≥5, <10 million yen | -2.80 [ -7.08 to 1.47] | | 0.19 | | 3.54 [-4.29 to 11.36] | | 0.36 | | -4.59 [-12.51 to 3.32] | | 0.24 | -4.15 [-13.03 to 4.72] | 0.34 | -4.68 [-14.42 to 5.07] | 0.33 |  |  |  |  |  |  |  |  |  |
| ≥10 million yen | -1.19 [-6.82 to 4.44] | | 0.66 | | 3.84 [-7.32 to 15.00] | | 0.48 | | -4.79 [-16.72 to 7.14] | | 0.41 | -2.64 [-15.44 to 10.17] | 0.67 | 0.93 [-10.59 to 12.45] | 0.87 |  |  |  |  |  |  |  |  |  |
| Education  (vs. up to elementary, middle school) | Ref. | |  | | Ref. | |  | | Ref. | |  | Ref. |  | Ref. |  |  |  |  |  |  |  |  |  |  |
| Up to high school, junior college | -1.00 [-4.83 to 2.83] | | 0.59 | | 13.64 [-6.26 to 33.54] | | 0.17 | | -11.60 [-22.01 to -1.18] | | 0.031 | -3.60 [-16.07 to 8.86] | 0.55 | -1.76 [-20.07 to 16.55] | 0.84 |  |  |  |  |  |  |  |  |  |
| Up to university, graduate school | -2.03 [-5.52 to 1.45] | | 0.24 | | 6.05 [-9.54 to 21.63] | | 0.43 | | -8.82 [-19.19 to 1.55] | | 0.091 | -2.37 [-16.11 to 11.38] | 0.72 | -5.27 [-19.68 to 9.15] | 0.46 |  |  |  |  |  |  |  |  |  |
| Factors of doctor |  | |  | |  | |  | |  | |  |  |  |  |  |  |  |  |  |  |  |  |  |  |
| Dr. age | -0.16 [-0.40 to 0.076] | | 0.17 | | -0.61 [-1.04 to -0.18] | | 0.008 | | 0.33 [-0.27 to 0.94] | | 0.27 | -0.024 [-0.52 to 0.47] | 0.92 | -0.22 [-0.89 to 0.46] | 0.51 |  |  |  |  |  |  |  |  |  |
| Dr. sex (female) | 0.43 [-4.69 to 5.54] | | 0.87 | | 11.37 [4.64 to 18.09] | | 0.002 | | -6.18 [-12.59 to 0.24] | | 0.058 | -1.96 [-9.27 to 5.35] | 0.58 | 1.10 [-10.65 to 12.84] | 0.85 |  |  |  |  |  |  |  |  |  |
| Baseline subdomain | -0.48 [-0.57 to -0.40] | | <0.001 | | -0.35 [-0.45 to -0.25] | | <0.001 | | -0.42 [-0.57 to -0.28] | | <0.001 | -0.51 [-0.64 to -0.38] | <0.001 | -0.51 [-0.59 to -0.43] | <0.001 |  |  |  |  |  |  |  |  |  |

^a^ At least one of the following: cyclophosphamide, mycophenolate mofetil, mizoribine, methotrexate, azathioprine, tacrolimus, cyclosporine, rituximab, or belimumab.

*SELENA-SLEDAI*, SELENA-Systemic Lupus Erythematosus Disease Activity Index; *SDI*, Systemic Lupus International Collaborating Clinics/American College of Rheumatology Damage Index.

**Supplemental Table 2 Comparison of the change in SDM-Q-9 to the change in LupusPRO in one year. (complete data)**

| **Variable** | **Change of Total HRQoL (n=277)** | | **Change of Lupus Symptoms (n=303)** | | **Change of Cognition**  **(n=305)** | | **Change of Lupus Medication**  **(n=314)** | | **Change of Procreation**  **(n=296)** | | **Change of Physical Health**  **(n=314)** | | **Change of Pain vitality**  **(n=310)** | | **Change of Emotional Health**  **(n=312)** | | **Change of Body Image**  **(n=308)** | |  |
| --- | --- | --- | --- | --- | --- | --- | --- | --- | --- | --- | --- | --- | --- | --- | --- | --- | --- | --- | --- |
|  | **β[95%CI]** | **P-value** | **β[95%CI]** | **P-value** | **β[95%CI]** | **P-value** | **β[95%CI]** | **P-value** | **β[95%CI]** | **P-value** | **β[95%CI]** | **P-value** | **β[95%CI]** | **P-value** | **β[95%CI]** | **P-value** | **β[95%CI]** | **P-value** | |
| Change of SDM-Q-9 score | 0.068 [-0.039 to 0.17] | 0.2 | -0.017 [-0.22 to 0.18] | 0.86 | -0.014 [-0.16 to 0.13] | 0.85 | 0.14 [-0.028 to 0.30] | 0.098 | 0.019 [-0.079 to 0.12] | 0.69 | 0.067 [-0.12 to 0.26] | 0.48 | 0.038 [-0.16 to 0.23] | 0.69 | 0.081 [-0.042 to 0.20] | 0.19 | 0.14 [0.025 to 0.26] | 0.02 | |
| Age at baseline (years) | 0.0059 [-0.14 to 0.15] | 0.93 | -0.017 [-0.16 to 0.13] | 0.814 | 0.059 [-0.19 to 0.31] | 0.632 | -0.050 [-0.23 to 0.13] | 0.57 | 0.26 [0.052 to 0.47] | 0.017 | 0.043 [-0.043 to 0.13] | 0.31 | -0.10 [-0.26 to 0.058] | 0.2 | -0.10 [-0.26 to 0.052] | 0.18 | -0.19 [-0.48 to 0.11] | 0.21 | |
| Sex (female) | 0.40 [-3.16 to 3.96] | 0.82 | -1.85 [-7.63 to 3.92] | 0.512 | -1.57 [-8.50 to 5.37] | 0.644 | 4.08 [-3.1 to 11.31] | 0.26 | 0.50 [-5.98 to 6.98] | 0.87 | -3.35 [-9.33 to 2.63] | 0.26 | 1.65 [-4.05 to 7.34] | 0.56 | 0.0092 [-5.34 to 5.36] | 1 | -2.77 [-8.71 to 3.16] | 0.34 | |
| Disease duration (year) | 0.064 [-0.075 to 0.20] | 0.35 | -0.025 [-0.23 to 0.18] | 0.81 | -0.022 [-0.31 to 0.26] | 0.88 | 0.19 [-0.13 to 0.51] | 0.23 | -0.024 [-0.19 to 0.14] | 0.76 | 0.032 [-0.19 to 0.26] | 0.77 | 0.11 [-0.17 to 0.40] | 0.42 | 0.057 [-0.19 to 0.31] | 0.64 | 0.14 [-0.11 to 0.38] | 0.27 | |
| SLEDAI | -0.17 [-0.63 to 0.29] | 0.45 | -0.087 [-0.82 to 0.65] | 0.81 | -0.24 [-1.13 to 0.65] | 0.59 | -0.38 [-1.18 to 0.42] | 0.33 | 0.10 [-0.32 to 0.53] | 0.61 | -0.13 [-0.51 to 0.25] | 0.47 | -0.0048 [-0.50 to 0.49] | 0.98 | -0.50 [-1.15 to 0.15] | 0.13 | 0.12 [-0.34 to 0.59] | 0.59 | |
| SDI | -0.38 [-1.48 to 0.72] | 0.48 | -1.03 [-3.05 to 0.98] | 0.3 | 0.86 [-1.95 to 3.67] | 0.53 | -2.80 [-4.71 to -0.90] | 0.006 | 0.31 [-0.84 to 1.47] | 0.58 | -1.41 [-2.34 to -0.48] | 0.005 | -0.24 [-1.43 to 0.96] | 0.68 | -0.37 [-2.11 to 1.36] | 0.66 | -0.60 [-2.24 to 1.04] | 0.46 | |
| Glucocorticoid dose (mg/day) | 0.090 [-0.38 to 0.56] | 0.69 | 0.30 [-0.30 to 0.90] | 0.31 | -0.27 [-0.74 to 0.19] | 0.24 | -0.11 [-0.67 to 0.46] | 0.7 | 0.073 [-0.43 to 0.57] | 0.77 | 0.17 [-0.13 to 0.46] | 0.26 | -0.044 [-0.53 to 0.45] | 0.86 | 0.17 [-0.47 to 0.81] | 0.58 | -0.16 [-0.73 to 0.41] | 0.57 | |
| Immunosuppressants^a^ | -1.26 [-3.36 to 0.83] | 0.22 | -0.080 [-5.82 to 5.66] | 0.98 | -1.66 [-6.41 to 3.08] | 0.48 | -1.17 [-7.79 to 5.44] | 0.72 | -1.61 [-5.51 to 2.29] | 0.4 | -1.53 [-6.17 to 3.11] | 0.5 | -0.31 [-5.38 to 4.77] | 0.9 | -2.41 [-6.44 to 1.62] | 0.23 | -5.57 [-9.71 to -1.43] | 0.011 | |
| Hydroxychloroquine | 1.45 [0.028 to 2.86] | 0.046 | 2.39 [-2.43 to 7.21] | 0.31 | 1.27 [-2.62 to 5.16] | 0.51 | 2.26 [-2.49 to 7.01] | 0.33 | -1.01 [-4.76 to 2.75] | 0.58 | 4.69 [2.26 to 7.13] | 0.001 | 0.59 [-3.05 to 4.23] | 0.74 | -2.12 [-5.72 to 1.47] | 0.23 | -3.05 [-7.80 to 1.70] | 0.2 | |
| Married state | 0.98 [-3.51 to 5.48] | 0.65 | 1.78 [-5.10 to 8.66] | 0.6 | 1.00 [-6.02 to 8.02] | 0.77 | 2.75 [-5.79 to 11.29] | 0.51 | 0.17 [-5.18 to 5.53] | 0.95 | 3.68 [-0.20 to 7.55] | 0.062 | 2.05 [-2.92 to 7.02] | 0.4 | -1.78 [-5.84 to 2.28] | 0.37 | -2.32 [-7.58 to 2.93] | 0.37 | |
| Annual income  (vs. <2.5 million yen) | Ref. |  | Ref. |  | Ref. |  | Ref. |  | Ref. |  | Ref. |  | Ref. |  | Ref. |  | Ref. |  | |
| ≥2.5, <5 million yen | 4.24 [0.22 to 8.26] | 0.04 | 4.59 [-2.75 to 11.93] | 0.21 | 11.50 [4.02 to 18.99] | 0.005 | 2.77 [-4.78 to 10.31] | 0.45 | 5.63 [-2.08 to 13.34] | 0.14 | -0.83 [-6.41 to 4.75] | 0.76 | 2.61 [-5.51 to 10.72] | 0.51 | 7.46 [0.77 to 14.15] | 0.031 | 5.19 [-2.03 to 12.42] | 0.15 | |
| ≥5, <10 million yen | 5.29 [1.59 to 8.99] | 0.008 | 4.12 [-3.26 to 11.49] | 0.26 | 11.14 [4.12 to 18.17] | 0.004 | 8.16 [-0.0076 to 16.33] | 0.05 | 6.53 [-0.73 to 13.78] | 0.075 | 3.30 [-1.53 to 8.14] | 0.17 | 3.20 [-3.40 to 9.80] | 0.32 | 9.36 [2.96 to 15.75] | 0.007 | 3.65 [-4.73 to 12.02] | 0.38 | |
| ≥10 million yen | 6.62 [2.23 to 11.01] | 0.006 | 4.91 [-3.36 to 13.18] | 0.23 | 13.76 [5.61 to 21.91] | 0.002 | 9.60 [-1.59 to 20.79] | 0.088 | 4.06 [-4.81 to 12.93] | 0.35 | 2.31 [-2.46 to 7.08] | 0.32 | 3.93 [-5.00 to 12.86] | 0.37 | 15.64 [6.88 to 24.40] | 0.001 | 2.63 [-6.89 to 12.15] | 0.57 | |
| Education  (vs. up to elementary, middle school) | Ref. |  | Ref. |  | Ref. |  | Ref. |  | Ref. |  | Ref. |  | Ref. |  | Ref. |  | Ref. |  | |
| Up to high school, junior college | -0.31 [-8.97 to 8.34] | 0.94 | -3.64 [-17.88 to 10.59] | 0.6 | 5.67 [-11.79 to 23.13] | 0.51 | -4.12 [-21.87 to 13.64] | 0.63 | -4.90 [-23.04 to 13.24] | 0.58 | -1.27 [-10.76 to 8.22] | 0.78 | -2.70 [-11.06 to 5.65] | 0.51 | -2.37 [-13.95 to 9.21] | 0.68 | 10.26 [-4.44 to 24.96] | 0.16 | |
| Up to university, graduate school | -1.93 [-10.97 to 7.12] | 0.66 | -4.01 [-18.76 to 10.74] | 0.58 | 2.75 [-15.45 to 20.95] | 0.76 | -4.99 [-23.62 to 13.64] | 0.58 | -3.46 [-21.05 to 14.12] | 0.69 | -4.30 [-13.22 to 4.63] | 0.33 | -6.99 [-16.84 to 2.85] | 0.15 | -7.21 [-18.03 to 3.61] | 0.18 | 5.08 [-9.18 to 19.34] | 0.47 | |
| Factors of doctor |  |  |  |  |  |  |  |  |  |  |  |  |  |  |  |  |  |  | |
| Dr. age | -0.19 [-0.43 to 0.053] | 0.12 | -0.24 [-0.68 to 0.21] | 0.28 | -0.45 [-0.94 to 0.048] | 0.074 | -0.30 [-0.95 to 0.36] | 0.36 | 0.084 [-0.44 to 0.60] | 0.74 | 0.0069 [-0.19 to 0.21] | 0.94 | -0.41 [-0.81 to -0.021] | 0.04 | -0.14 [-0.44 to 0.16] | 0.34 | 0.052 [-0.57 to 0.67] | 0.87 | |
| Dr. sex (female) | 6.25 [2.86 to 9.65] | 0.001 | 6.75 [-0.17 to 13.66] | 0.055 | 10.29 [3.78 to 16.80] | 0.003 | 9.28 [-0.79 to 19.36] | 0.069 | 0.67 [-2.99 to 4.33] | 0.71 | 6.03 [3.63 to 8.42] | <0.001 | 6.24 [1.072 to 11.42] | 0.02 | 4.98 [-0.39 to 10.34] | 0.067 | 5.89 [-5.23 to 17.01] | 0.28 | |
| Baseline subdomain | -0.28 [-0.36 to -0.20] | <0.001 | -0.42 [-0.59 to -0.25] | <0.001 | -0.47 [-0.58 to -0.35] | <0.001 | -0.51 [-0.63 to -0.40] | <0.001 | -0.44 [-0.62 to -0.26] | <0.001 | -0.37 [-0.51 to -0.23] | <0.001 | -0.39 [-0.47 to -0.32] | <0.001 | -0.27 [-0.38 to -0.16] | <0.001 | -0.35 [-0.43 to -0.28] | <0.001 | |

| **Variable** | **Change of Total N-HRQoL**  **(n=295)** | | **Change of Desires-Goals**  **(n=308)** | | **Change of Social Support**  **(n=312)** | | **Change of Coping**  **(n=307)** | | **Change of**  **Satisfaction with care**  **(n=304)** | |
| --- | --- | --- | --- | --- | --- | --- | --- | --- | --- | --- |
|  | **β[95%CI]** | **P-value** | **β[95%CI]** | **P-value** | **β[95%CI]** | **P-value** | **β[95%CI]** | **P-value** | **β[95%CI]** | **P-value** |
| Change of SDM-Q-9 score | 0.013 [-0.036 to 0.062] | 0.58 | 0.11 [-0.018 to 0.23] | 0.088 | -0.10 [-0.20 to -0.0075] | 0.036 | -0.046 [-0.19 to 0.095] | 0.51 | 0.20 [0.063 to 0.33] | 0.006 |
| Age at baseline (years) | -0.029 [-0.13 to 0.076] | 0.57 | -0.039 [-0.23 to 0.15] | 0.67 | -0.0086 [-0.19 to 0.18] | 0.92 | 0.18 [-0.024 to 0.39] | 0.08 | -0.26 [-0.70 to 0.18] | 0.23 |
| Sex (female) | -0.26 [-3.81 to 3.29] | 0.88 | 1.45 [-4.81 to 7.72] | 0.64 | 4.99 [-0.95 to 10.93] | 0.095 | -1.91 [-8.88 to 5.05] | 0.58 | -5.42 [-17.51 to 6.67] | 0.36 |
| Disease duration (year) | 0.061 [-0.067 to 0.19] | 0.33 | 0.11 [-0.15 to 0.36] | 0.39 | -0.18 [-0.41 to 0.060] | 0.14 | -0.13 [-0.33 to 0.060] | 0.17 | 0.31 [0.00018 to 0.61] | 0.05 |
| SLEDAI | 0.14 [-0.29 to 0.58] | 0.5 | -0.27 [-0.69 to 0.15] | 0.2 | -0.19 [-0.88 to 0.51] | 0.59 | 0.50 [-0.45 to 1.45] | 0.29 | 0.48 [-0.15 to 1.12] | 0.13 |
| SDI | -0.29 [-1.22 to 0.64] | 0.52 | 0.24 [-1.56 to 2.04] | 0.79 | 1.21 [-1.16 to 3.59] | 0.3 | -0.51 [-1.87 to 0.85] | 0.45 | -1.59 [-3.63 to 0.45] | 0.12 |
| Glucocorticoid dose (mg/day) | -0.074 [-0.29 to 0.14] | 0.49 | 0.31 [-0.31 to 0.93] | 0.32 | -0.45 [-0.83 to -0.077] | 0.021 | -0.0054 [-0.50 to 0.49] | 0.98 | 0.051 [-0.30 to 0.40] | 0.77 |
| Immunosuppressants^a^ | 2.58 [0.50 to 4.66] | 0.017 | -0.91 [-5.60 to 3.78] | 0.69 | 2.66 [-1.64 to 6.95] | 0.21 | 1.30 [-1.73 to 4.32] | 0.38 | 5.66 [0.613 to 10.71] | 0.03 |
| Hydroxychloroquine | 0.014 [-2.60 to 2.63] | 0.99 | 0.40 [-2.78 to 3.58] | 0.8 | 0.30 [-4.58 to 5.18] | 0.9 | 1.94 [-6.69 to 10.56] | 0.65 | -3.38 [-7.89 to 1.129] | 0.13 |
| Married state | -0.88 [-3.84 to 2.08] | 0.54 | 3.73 [-2.84 to 10.29] | 0.25 | -2.07 [-7.43 to 3.29] | 0.43 | 0.58 [-4.51 to 5.68] | 0.82 | -4.99 [-11.55 to 1.57] | 0.13 |
| Annual income  (vs. <2.5 million yen) | Ref. |  | Ref. |  | Ref. |  | Ref. |  | Ref. |  |
| ≥2.5, <5 million yen | -1.10 [-5.29 to 3.08] | 0.59 | 0.94 [-7.33 to 9.20] | 0.82 | -3.76 [-11.99 to 4.46] | 0.35 | -2.53 [-9.60 to 4.55] | 0.46 | 1.83 [-6.36 to 10.01] | 0.65 |
| ≥5, <10 million yen | -2.43 [-6.53 to 1.67] | 0.23 | 3.54 [-4.39 to 11.47] | 0.36 | -4.78 [-12.51 to 2.96] | 0.21 | -3.92 [-12.38 to 4.53] | 0.35 | -3.74 [-12.65 to 5.17] | 0.39 |
| ≥10 million yen | -1.14 [-7.11 to 4.84] | 0.7 | 3.44 [-7.25 to 14.12] | 0.51 | -4.30 [-16.41 to 7.80] | 0.47 | -2.61 [-15.61 to 10.39] | 0.68 | 0.76 [-10.96 to 12.47] | 0.89 |
| Education  (vs. up to elementary, middle school) | Ref. |  | Ref. |  | Ref. |  | Ref. |  | Ref. |  |
| Up to high school, junior college | -0.93 [-4.82 to 2.96] | 0.62 | 14.16 [-6.71 to 35.02] | 0.17 | -12.19 [-22.35 to -2.03] | 0.021 | -4.73 [-17.27 to 7.80] | 0.44 | -0.98 [-16.72 to 14.77] | 0.9 |
| Up to university, graduate school | -2.41 [-6.24 to 1.41] | 0.2 | 6.44 [-9.80 to 22.68] | 0.42 | -9.31 [-19.63 to 1.01] | 0.075 | -3.88 [-17.78 to 10.01] | 0.57 | -5.98 [-17.72 to 5.76] | 0.3 |
| Factors of doctor |  |  |  |  |  |  |  |  |  |  |
| Dr. age | -0.066 [-0.30 to 0.17] | 0.56 | -0.56 [-1.00 to -0.13] | 0.012 | 0.32 [-0.19 to 0.82] | 0.2 | 0.076 [-0.33 to 0.48] | 0.7 | -0.010 [-0.79 to 0.77] | 0.98 |
| Dr. sex (female) | 1.91 [-2.66 to 6.49] | 0.39 | 11. 90 [5.34 to 18.45] | 0.001 | -6.20 [-13.32 to 0.91] | 0.084 | -0.18 [-7.12 to 6.77] | 0.96 | 4.49 [-5.07 to 14.06] | 0.34 |
| Baseline subdomain | -0.42 [-0.50 to -0.35] | <0.001 | -0.34 [-0.44 to -0.25] | <0.001 | -0.42 [-0.58 to -0.26] | <0.001 | -0.50 [-0.63 to -0.37] | <0.001 | -0.42 [-0.47 to -0.37] | <0.001 |

^a^At least one of the following: cyclophosphamide, mycophenolate mofetil, mizoribine, methotrexate, azathioprine, tacrolimus, cyclosporine, rituximab, or belimumab.

SELENA-SLEDAI, SELENA-Systemic Lupus Erythematosus Disease Activity Index; SDI, Systemic Lupus International Collaborating Clinics/American College of Rheumatology Damage Index.

**Supp****lemental Table 3 Full data on the changes in SDM after one year in high and low SDM groups and their impact on LupusPRO.**

| **Variable** | **Total HRQoL**  **(n=277)** | | **Lupus symptoms**  **(n=303)** | | **Cognition (n=305)** | | **Lupus Medications**  **(n=314)** | | **Procreation**  **(n=296)** | | **Physical Health**  **(n=314)** | | **Pain Vitality**  **(n=310)** | | **Emotional Health**  **(n=312)** | | **Body Image**  **(n=308)** | |  |
| --- | --- | --- | --- | --- | --- | --- | --- | --- | --- | --- | --- | --- | --- | --- | --- | --- | --- | --- | --- |
|  | **β[95%CI]** | **P-value** | **β[95%CI]** | **P-value** | **β[95%CI]** | **P-value** | **β[95%CI]** | **P-value** | **β[95%CI]** | **P-value** | **β[95%CI]** | **P-value** | **β[95%CI]** | **P-value** | **β[95%CI]** | **P-value** | **β[95%CI]** | **P-value** | |
| Change of SDM-Q-9 (vs. LD group) | Ref. |  | Ref. |  | Ref. |  | Ref. |  | Ref. |  | Ref. |  | Ref. |  | Ref. |  | Ref. |  | |
| LI group | -0.35 [-4.92 to 4.22] | 0.88 | -7.02 [-14.69 to 0.65] | 0.071 | -3.01 [-11.48 to 5.46] | 0.47 | -3.18 [-9.32 to 2.96] | 0.3 | 4.79 [-1.13 to 10.71] | 0.11 | -0.63 [-3.59 to 2.33] | 0.67 | -3.81 [-10.16 to 2.55] | 0.23 | -4.32 [-9.69 to 1.05] | 0.11 | 2.79 [-4.52 to 10.11] | 0.44 | |
| HD group | 0.0047 [-4.19 to 4.20] | 1.0 | -3.50 [-9.96 to 2.96] | 0.27 | -1.86 [-7.98 to 4.26] | 0.54 | -1.26 [-6.89 to 4.37] | 0.65 | 7.15 [3.75 to 10.55] | <0.001 | -1.59 [-5.85 to 2.66] | 0.45 | -6.15 [-10.29 to -2.00] | 0.006 | -0.19 [-5.70 to 5.31] | 0.94 | 3.59 [-5.99 to 13.17] | 0.45 | |
| HI group | 1.17 [-2.04 to 4.38] | 0.45 | -4.99 [-10.54 to 0.57] | 0.076 | 1.22 [-4.18 to 6.62] | 0.64 | 2.11 [-1.66 to 5.89] | 0.26 | 7.10 [2.41 to 11.79] | 0.005 | -1.33 [-5.12 to 2.46] | 0.47 | -1.32 [-5.73 to 3.10] | 0.54 | 2.44 [-2.35 to 7.23] | 0.3 | 4.24 [-2.36 to 10.84] | 0.2 | |
| Age at baseline (years) | 0.021 [-0.12 to 0.16] | 0.75 | -0.022 [-0.16 to 0.11] | 0.74 | 0.070 [-0.18 to 0.32] | 0.56 | -0.030 [-0.24 to 0.16] | 0.74 | 0.27 [0.067 to 0.48] | 0.012 | 0.047 [-0.041 to 0.14] | 0.28 | -0.098 [-0.25 to 0.051] | 0.19 | -0.073 [-0.25 to 0.098] | 0.38 | -0.16 [-0.45 to 0.13] | 0.27 | |
| Sex (female) | -0.057 [-3.76 to 3.64] | 0.97 | -2.70 [-8.44 to 3.04] | 0.34 | -1.87 [-8.66 to 4.92] | 0.57 | 2.93 [-4.28 to 10.13] | 0.41 | 0.62 [-5.52 to 6.77] | 0.84 | -3.73 [-9.36 to 1.90] | 0.18 | 1.09 [-4.18 to 6.37] | 0.67 | -0.95 [-6.59 to 4.70] | 0.73 | -3.30 [-9.11 to 2.50] | 0.25 | |
| Disease duration (year) | 0.074 [-0.085 to 0.23] | 0.34 | 0.0062 [-0.25 to 0.26] | 0.96 | 0.0096 [-0.26 to 0.28] | 0.94 | 0.23 [-0.13 to 0.58] | 0.2 | -0.025 [-0.18 to 0.13] | 0.75 | 0.036 [-0.20 to 0.27] | 0.76 | 0.13 [-0.19 to 0.45] | 0.403 | 0.10 [-0.16 to 0.37] | 0.43 | 0.145 [-0.13 to 0.42] | 0.28 | |
| SLEDAI | -0.16 [-0.62 to 0.31] | 0.49 | -0.060 [-0.77 to 0.65] | 0.86 | -0.25 [-1.16 to 0.66] | 0.58 | -0.35 [-1.16 to 0.45] | 0.37 | 0.071 [-0.34 to 0.48] | 0.72 | -0.11 [-0.46 to 0.24] | 0.51 | 0.0089 [-0.49 to 0.50] | 0.97 | -0.51 [-1.17 to 0.15] | 0.12 | 0.14 [-0.33 to 0.62] | 0.54 | |
| SDI | -0.47 [-1.61 to 0.68] | 0.4 | -1.16 [-3.23 to 0.91] | 0.26 | 0.72 [-2.01 to 3.45] | 0.59 | -2.98 [-4.83 to -1.12] | 0.003 | 0.25 [-0.81 to 1.30] | 0.64 | -1.46 [-2.38 to -0.54] | 0.003 | -0.37 [-1.49 to 0.76] | 0.51 | -0.58 [-2.28 to 1.11] | 0.48 | -0.66 [-2.28 to 0.95] | 0.4 | |
| Glucocorticoid dose (mg/day) | 0.093 [-0.36 to 0.55] | 0.67 | 0.30 [-0.30 to 0.91] | 0.31 | -0.27 [-0.73 to 0.18] | 0.23 | -0.10 [-0.66 to 0.45] | 0.7 | 0.085 [-0.38 to 0.55] | 0.71 | 0.17 [-0.12 to 0.45] | 0.24 | -0.043 [-0.52 to 0.43] | 0.85 | 0.17 [-0.44 to 0.78] | 0.57 | -0.16 [-0.71 to 0.40] | 0.56 | |
| Immunosuppressants^a^ | -1.40 [-3.64 to 0.84] | 0.21 | -0.30 [-5.96 to 5.36] | 0.91 | -1.68 [-6.43 to 3.07] | 0.47 | -1.53 [-8.45 to 5.40] | 0.65 | -1.71 [-5.90 to 2.48] | 0.41 | -1.58 [-6.04 to 2.87] | 0.47 | -0.22 [-5.18 to 4.74] | 0.93 | -2.75 [-6.89 to 1.39] | 0.18 | -5.76 [-10.01 to -1.52] | 0.01 | |
| Hydroxychloroquine | 1.38 [-0.15 to 2.90] | 0.074 | 2.462 [-3.16 to 8.09] | 0.37 | 1.60 [-2.20 to 5.40] | 0.39 | 2.32 [-2.86 to 7.50] | 0.36 | -1.14 [-4.83 to 2.55] | 0.53 | 4.64 [2.22 to 7.05] | 0.001 | 0.87 [-2.42 to 4.15] | 0.59 | -1.96 [-5.40 to 1.48] | 0.25 | -3.37 [-8.74 to 1.99] | 0.21 | |
| Married state | 0.89 [-3.43 to 5.20] | 0.67 | 1.41 [-5.52 to 8.33] | 0.68 | 1.17 [-5.73 to 8.07] | 0.73 | 2.41 [-6.30 to 11.13] | 0.57 | 0.43 [-4.90 to 5.75] | 0.87 | 3.43 [-0.36 to 7.22] | 0.074 | 1.71 [-3.46 to 6.89] | 0.5 | -1.95 [-6.21 to 2.31] | 0.35 | -2.13 [-7.21 to 2.96] | 0.4 | |
| Annual income  (vs. <2.5 million yen) | Ref. |  | Ref. |  | Ref. |  |  |  | Ref. |  |  |  | Ref. |  | Ref. |  | Ref. |  | |
| ≥2.5, <5 million yen | 4.26 [0.30 to 8.23] | 0.036 | 4.74 [-2.76 to 12.23] | 0.2 | 10.97 [3.93 to 18.01] | 0.004 | 2.91 [-4.78 to 10.60] | 0.44 | 5.40 [-2.66 to 13.46] | 0.18 | -0.70 [-6.03 to 4.63] | 0.79 | 2.80 [-5.27 to 10.88] | 0.48 | 7.38 [1.15 to 13.61] | 0.023 | 5.26 [-2.01 to 12.53] | 0.15 | |
| ≥5, <10 million yen | 5.15 [1.58 to 8.72] | 0.008 | 3.99 [-3.23 to 11.21] | 0.26 | 10.74 [3.62 to 17.87] | 0.005 | 7.67 [-0.57 to 15.90] | 0.066 | 5.82 [-1.50 to 13.15] | 0.11 | 3.47 [-1.35 to 8.29] | 0.15 | 3.69 [-2.95 to 10.33] | 0.26 | 8.94 [2.64 to 15.23] | 0.008 | 3.44 [-5.22 to 12.10] | 0.42 | |
| ≥10 million yen | 6.50 [2.34 to 10.66] | 0.005 | 5.26 [-3.12 to 13.64] | 0.2 | 12.96 [5.23 to 20.69] | 0.003 | 9.09 [-2.83 to 21.01] | 0.13 | 3.19 [-5.54 to 11.93] | 0.45 | 2.55 [-2.20 to 7.29] | 0.27 | 3.60 [-4.97 to 12.17] | 0.39 | 15.45 [5.92 to 24.99] | 0.003 | 2.61 [-6.35 to 11.58] | 0.55 | |
| Education  (vs. up to elementary, middle school) | Ref. |  | Ref. |  | Ref. |  |  |  | Ref. |  |  |  | Ref. |  | Ref. |  | Ref. |  | |
| Up to high school, junior college | -0.90 [-9.58 to 7.78] | 0.83 | -3.93 [-18.49 to 10.62] | 0.58 | 5.11 [-11.76 to 21.98] | 0.54 | -3.92 [-21.28 to 13.44] | 0.64 | -3.89 [-21.08 to 13.30] | 0.64 | -1.67 [-10.98 to 7.64] | 0.71 | -3.02 [-11.16 to 5.13] | 0.45 | -2.68 [-13.81 to 8.45] | 0.62 | 9.60 [-5.67 to 24.87] | 0.21 | |
| Up to university, graduate school | -2.42 [-11.36 to 6.51] | 0.58 | -4.54 [-19.36 to 10.29] | 0.53 | 2.15 [-15.42 to 19.72] | 0.8 | -4.90 [-23.03 to 13.23] | 0.58 | -2.18 [-19.25 to 14.89] | 0.79 | -4.87 [-13.73 to 3.98] | 0.27 | -7.54 [-18.09 to 3.01] | 0.15 | -7.61 [-18.00 to 2.78] | 0.14 | 4.64 [-10.19 to 19.47] | 0.52 | |
| Factors of doctor |  |  |  |  |  |  |  |  |  |  |  |  |  |  |  |  |  |  | |
| Dr age | -0.23 [-0.47 to 0.017] | 0.067 | -0.267 [-0.72 to 0.19] | 0.24 | -0.49 [-1.01 to 0.02] | 0.059 | -0.40 [-1.10 to 0.30] | 0.25 | 0.065 [-0.35 to 0.48] | 0.75 | -0.0017 [-0.21 to 0.21] | 0.99 | -0.44 [-0.83 to -0.053] | 0.028 | -0.25 [-0.56 to 0.063] | 0.11 | -0.017 [-0.63 to 0.60] | 0.96 | |
| Dr sex (female) | 5.84 [1.85 to 9.82] | 0.006 | 6.98 [0.62 to 13.34] | 0.033 | 10.37 [2.52 to 18.22] | 0.012 | 8.42 [-3.10 to 19.94] | 0.14 | -0.89 [-5.11 to 3.33] | 0.67 | 6.24 [3.55 to 8.93] | 0 | 7.26 [1.90 to 12.61] | 0.01 | 3.83 [-2.37 to 10.036] | 0.21 | 4.52 [-6.66 to 15.70] | 0.41 | |
| Baseline subdomain | -0.29 [-0.37 to -0.21] | <0.001 | -0.42 [-0.58 to -0.25] | <0.001 | -0.47 [-0.58 to -0.36] | <0.001 | -0.51 [-0.64 to -0.39] | <0.001 | -0.45 [-0.61 to -0.29] | <0.001 | -0.38 [-0.52 to -0.24] | <0.001 | -0.40 [-0.47 to -0.33] | <0.001 | -0.28 [-0.38 to -0.18] | <0.001 | -0.35 [-0.43 to -0.27] | <0.001 | |

| **Variable** | **Total N-HRQoL**  **(n=295)** | | **Desires-Goals**  **(n=308)** | | **Social Support**  **(n=312)** | | **Coping**  **(n=307)** | | **Satisfaction with care**  **(n=304)** | |
| --- | --- | --- | --- | --- | --- | --- | --- | --- | --- | --- |
|  | **β[95%CI]** | **P-value** | **β[95%CI]** | **P-value** | **β[95%CI]** | **P-value** | **β[95%CI]** | **P-value** | **β[95%CI]** | **P-value** |
| Change of SDM-Q-9 (vs. LD group) | Ref. |  | Ref. |  | Ref. |  | Ref. |  | Ref. |  |
| LI group | 1.14 [-3.38 to 5.65] | 0.61 | -0.40 [-6.41 to 5.62] | 0.89 | -5.49 [-12.63 to 1.65] | 0.13 | 2.68 [-4.83 to 10.19] | 0.47 | 6.96 [0.064 to 13.85] | 0.048 |
| HD group | 4.06 [-1.94 to 10.06] | 0.18 | 1.12 [-6.76 to 9.00] | 0.77 | -4.07 [-12.80 to 4.66] | 0.35 | 3.77 [-5.57 to 13.12] | 0.41 | 13.75 [6.39 to 21.11] | 0.001 |
| HI group | 7.06 [2.47 to 11.65] | 0.004 | 2.94 [-2.26 to 8.15] | 0.25 | -2.88 [-12.97 to 7.21] | 0.56 | 9.18 [3.63 to 14.73] | 0.002 | 19.40 [12.25 to 26.55] | <0.001 |
| Age at baseline (years) | -0.0048 [-0.12 to 0.11] | 0.93 | -0.017 [-0.19 to 0.16] | 0.85 | -0.021 [-0.21 to 0.17] | 0.82 | 0.20 [0.0020 to 0.40] | 0.048 | -0.21 [-0.68 to 0.26] | 0.37 |
| Sex (female) | -0.30 [-3.70 to 3.09] | 0.86 | 0.73 [-6.28 to 7.75] | 0.83 | 4.84 [-1.18 to 10.85] | 0.11 | -1.44 [-8.46 to 5.59] | 0.68 | -6.22 [-17.79 to 5.36] | 0.28 |
| Disease duration (year) | 0.065 [-0.078 to 0.21] | 0.36 | 0.14 [-0.13 to 0.40] | 0.31 | -0.17 [-0.40 to 0.070] | 0.16 | -0.13 [-0.35 to 0.097] | 0.25 | 0.30 [-0.060 to 0.65] | 0.099 |
| SLEDAI | 0.11 [-0.33 to 0.56] | 0.6 | -0.25 [-0.66 to 0.16] | 0.22 | -0.18 [-0.91 to 0.55] | 0.62 | 0.44 [-0.53 to 1.41] | 0.36 | 0.46 [-0.16 to 1.08] | 0.14 |
| SDI | -0.28 [-1.24 to 0.69] | 0.56 | 0.058 [-1.66 to 1.78] | 0.95 | 1.12 [-1.25 to 3.49] | 0.34 | -0.45 [-1.72 to 0.82] | 0.47 | -1.33 [-3.77 to 1.10] | 0.27 |
| Glucocorticoid dose (mg/day) | -0.057 [-0.22 to 0.11] | 0.48 | 0.29 [-0.33 to 0.92] | 0.34 | -0.46 [-0.84 to -0.073] | 0.022 | 0.013 [-0.46 to 0.49] | 0.95 | 0.090 [-0.15 to 0.33] | 0.44 |
| Immunosuppressants^a^ | 2.42 [0.39 to 4.44] | 0.021 | -1.16 [-6.05 to 3.74] | 0.63 | 2.68 [-1.84 to 7.21] | 0.23 | 1.19 [-1.65 to 4.02] | 0.39 | 5.62 [0.66 to 10.58] | 0.028 |
| Hydroxychloroquine | 0.020 [-2.46 to 2.50] | 0.99 | 0.36 [-2.81 to 3.52] | 0.82 | 0.63 [-4.66 to 5.93] | 0.81 | 2.24 [-6.05 to 10.54] | 0.58 | -3.61 [-7.98 to 0.77] | 0.1 |
| Married state | -0.59 [-3.52 to 2.34] | 0.68 | 3.60 [-2.61 to 9.81] | 0.24 | -2.10 [-7.08 to 2.89] | 0.39 | 1.07 [-3.52 to 5.67] | 0.63 | -4.30 [-11.06 to 2.47] | 0.2 |
| Annual income  (vs. <2.5 million yen) | Ref. |  | Ref. |  | Ref. |  | Ref. |  | Ref. |  |
| ≥2.5, <5 million yen | -1.20 [-4.92 to 2.52] | 0.51 | 0.99 [-7.14 to 9.12] | 0.8 | -4.02 [-11.96 to 3.91] | 0.3 | -2.69 [-9.40 to 4.02] | 0.41 | 1.41 [-5.99 to 8.80] | 0.69 |
| ≥5, <10 million yen | -2.77 [-6.90 to 1.36] | 0.18 | 3.25 [-4.44 to 10.95] | 0.39 | -4.65 [-12.42 to 3.13] | 0.23 | -4.16 [-12.95 to 4.62] | 0.34 | -5.28 [-14.93 to 4.36] | 0.27 |
| ≥10 million yen | -1.59 [-7.81 to 4.63] | 0.6 | 3.38 [-7.19 to 13.96] | 0.51 | -4.88 [-16.79 to 7.02] | 0.4 | -3.48 [-17.10 to 10.13] | 0.6 | -0.60 [-13.38 to 12.18] | 0.92 |
| Education  (vs. up to elementary, middle school) | Ref. |  |  |  |  |  | Ref. |  | Ref. |  |
| Up to high school, junior college | -1.18 [-5.18 to 2.81] | 0.54 | 13.29 [-6.72 to 33.29] | 0.18 | -11.88 [-21.78 to -1.98] | 0.021 | -4.04 [-16.07 to 8.00] | 0.49 | -2.12 [-18.30 to 14.06] | 0.79 |
| Up to university, graduate school | -2.66 [-6.75 to 1.42] | 0.19 | 5.62 [-9.69 to 20.93] | 0.45 | -9.33 [-19.21 to 0.56] | 0.063 | -3.26 [-17.16 to 10.64] | 0.63 | -6.48 [-19.65 to 6.70] | 0.32 |
| Factors of doctor |  |  |  |  |  |  |  |  |  |  |
| Dr age | -0.13 [-0.36 to 0.090] | 0.23 | -0.64 [-1.05 to -0.24] | 0.003 | 0.33 [-0.20 to 0.86] | 0.22 | 0.026 [-0.42 to 0.48] | 0.91 | -0.15 [-0.77 to 0.47] | 0.63 |
| Dr sex (female) | 0.81 [-3.99 to 5.60] | 0.73 | 10.95 [3.50 to 18.40] | 0.006 | -5.40 [-11.97 to 1.17] | 0.1 | -0.96 [-7.30 to 5.38] | 0.76 | 1.24 [-9.23 to 11.70] | 0.81 |
| Baseline subdomain | -0.47 [-0.54 to -0.40] | <0.001 | -0.35 [-0.45 to -0.25] | <0.001 | -0.42 [-0.58 to -0.26] | <0.001 | -0.51 [-0.64 to -0.38] | <0.001 | -0.49 [-0.56 to -0.43] | <0.001 |

^a^At least one of the following: cyclophosphamide, mycophenolate mofetil, mizoribine, methotrexate, azathioprine, tacrolimus, cyclosporine, rituximab, or belimumab.

LD, Decreasing SDM from Low Baseline; LI, Increasing SDM from Low Baseline; HD, Decreasing SDM from High Baseline; HI, Increasing SDM from High Baseline; SELENA-SLEDAI, SELENA-Systemic Lupus Erythematosus Disease Activity Index; SDI, Systemic Lupus International Collaborating Clinics/American College of Rheumatology Damage Index.


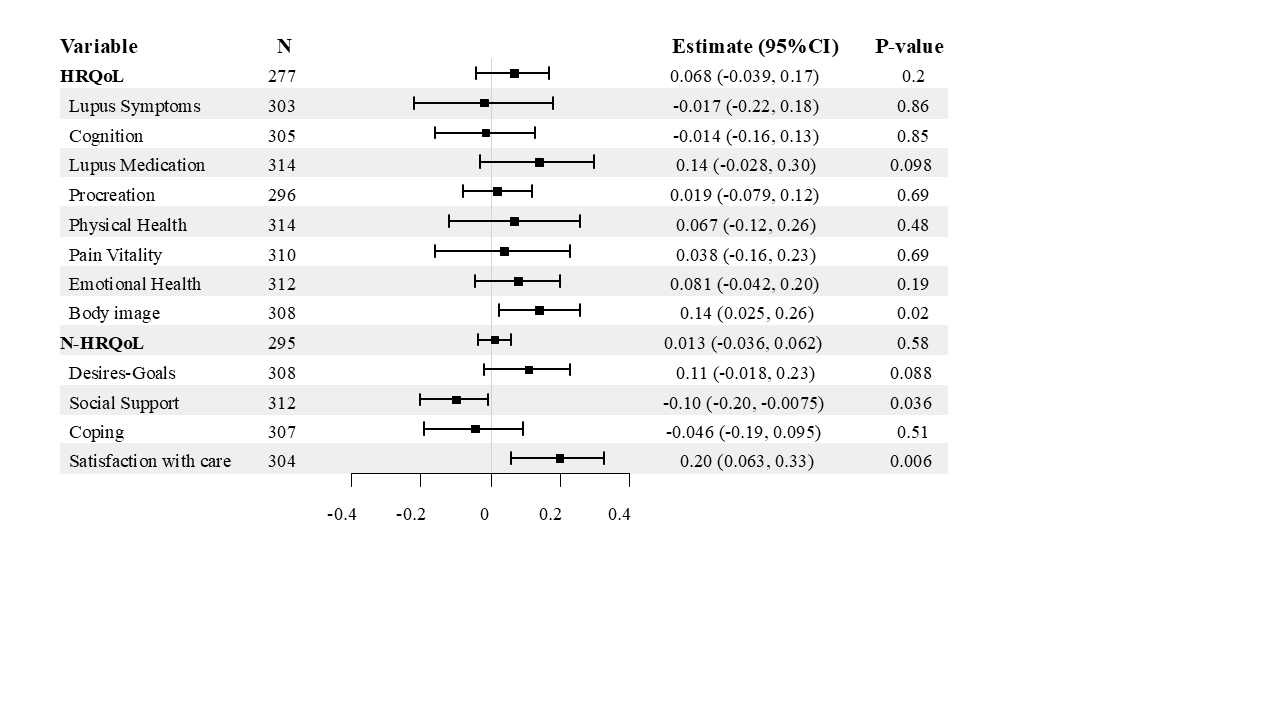


**Supplemental Figure 1**

A forest plot comparing the change in SDM-Q-9 to the change in LupusPRO after one year. We analyzed the impact of one-year changes in SDM-Q-9 scores on one-year changes in LupusPRO using general linear models. Full results are provided in Supplemental Table 2.


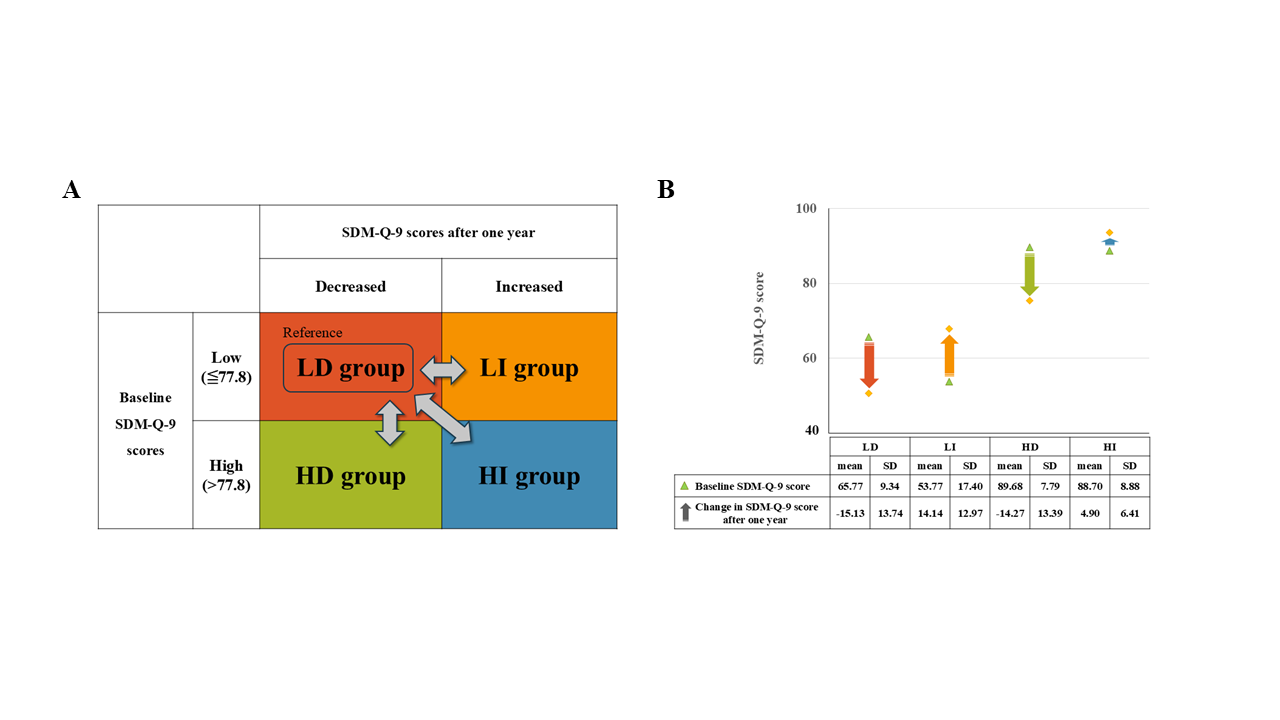


**Supplemental Figure 2**

A. Participants were classified into high-score and low-score groups based on the median baseline SDM-Q-9 score (77.8). Participants were further categorized within each group based on whether their SDM-Q-9 scores increased or decreased after one year compared to baseline. This classification resulted in four groups: LD group (low baseline score, decreased score), LI group (low baseline score, increased score), HD group (high baseline score, decreased score), and HI group (high baseline score, increased score). The LD group was used as the reference group for comparisons with the other three groups. B. Mean baseline SDM-Q-9 scores and their one-year mean changes across four groups. Triangles represent the mean baseline SDM-Q-9 score, and arrows indicate the mean change in SDM-Q-9 score after one year. Squares show the sum of the mean baseline SDM-Q-9 score and the mean one-year change in SDM-Q-9 score. The mean and standard deviation (SD) of both baseline and one-year change in SDM-Q-9 scores for each group are presented within the figure. LD, Decreasing SDM from Low Baseline; LI, Increasing SDM from Low Baseline; HD, Decreasing SDM from High Baseline; HI, Increasing SDM from High Baseline.
